# Effects of Nitric Oxide on the Activity of P2X and TRPV1 Receptors in Rat Meningeal Afferents of the Trigeminal Nerve

**DOI:** 10.3390/ijms24087519

**Published:** 2023-04-19

**Authors:** Kseniia Koroleva, Svetlana Svitko, Anton Ananev, Anastasiia Buglinina, Ksenia Bogatova, Olga Yakovleva, Dinara Nurmieva, Ilnar Shaidullov, Guzel Sitdikova

**Affiliations:** Department of Human and Animal Physiology, Institute of Fundamental Medicine and Biology, Kazan Federal University, 420008 Kazan, Russia

**Keywords:** nitric oxide, nitroglycerine, trigeminal nerve, migraine, TRPV1, P2X3 receptor, mast cell, S-nitrosylation

## Abstract

Nitric oxide is one of the endogenous molecules that play a key role in migraine. However, the interaction between NO and the main players in the nociceptive activity of the meningeal trigeminal afferents—TRPV1 and P2X3 receptors—remains unstudied. In the current project, the effects of acute and chronic NO administration on the activity of TRPV1 and P2X3 receptors in the peripheral afferents were studied using electrophysiological recording of action potentials of the trigeminal nerve in the rat hemiskull preparations. The data obtained indicate that exogenous and endogenous NO increased the activity of the trigeminal nerve independent on the inhibition of the TRPV1 and P2X3 receptors. The activity of the trigeminal nerve triggered by ATP changed neither in acute incubation in the NO donor—sodium nitroprusside (SNP) nor in the chronic nitroglycerine (NG)-induced migraine model. Moreover, the chronic NG administration did not increase in the number of degranulated mast cells in the rat meninges. At the same time, the capsaicin-induced activity of the trigeminal nerve was higher with chronic NO administration or after acute NO application, and these effects were prevented by N-ethylmaleimide. In conclusion, we suggested that NO positively modulates the activity of TRPV1 receptors by S-nitrosylation, which may contribute to the pro-nociceptive action of NO and underlie the sensitization of meningeal afferents in chronic migraine.

## 1. Introduction

Nitric oxide (NO) is a member of the gasotransmitters family produced in most of the mammalian tissues and involved in the regulation of a number of physiological functions [1,2,3,4,5]. Endogenously, NO is synthesized from L-arginine by nitric oxide synthases (NOS) including neuronal NO-synthase (nNOS), endothelial NO-synthase (eNOS), and inducible NO-synthase (iNOS) [3,6]. Among important physiological functions of NO is the regulation of vascular tone (including cerebral vessels) and nociception [6]. In this regard, NO is one of the endogenous molecules that play a key role in migraine, because its pathogenesis includes vascular as well as neuronal changes [7,8,9]. An important role of NO in migraine is confirmed by numerous experiment results. Thus, the injections of nitroglycerine (NG)—NO donors are widely used in human and animal migraine models; the specific NO-synthase inhibitors show an antinociceptive effect in patients suffering from migraine [10]. 

According to modern concepts, the activation of the peripheral afferents of the trigeminal nerve in meningeal tissues leads to the generation of a nociceptive signal and migraine headache [11,12]. The main sources of NO in the meninges are eNOS, localized in endotheliocytes of vessels, including the middle meningeal artery (MMA), and nNOS expressed in the thin branched nervous fibers located along the MMA. In addition, nNOS is found in part of the neurons of the trigeminal ganglion [13]. Previous studies indicate that NO is endogenously formed in the meningeal membranes and has pro-nociceptive effects in the afferents of the trigeminal nerve [14,15,16]. The known targets of NO include key elements underlying nociception such as TRPA1 and TRPV1 [17,18,19,20]. Activation of TRPA1 and TRPV1 in HEK cells has been shown to be mediated by S-nitrosylation through interaction between the NO and H2S, producing the nitroxyl ani-on—HNO− [19,21]. In DRG neurons, NO inhibited TRPV1 as a result of the activation of the sGC/cGMP/PKG signaling pathway [20].

Purinergic P2X receptors are widely expressed in meningeal tissues [17,22,23], and numerous data confirm their involvement in nociception [24,25,26,27]. The interplay of purinergic receptors (P2X and P2Y) and NO is described for a number of structures, such as the hypothalamus, brain stem, and vascular endothelium. ATP and UTP activate inotropic and metabotropic receptors during hypoxia, resulting in the activation of eNOS and vasodilatation [27,28,29]. In rat hippocampal neurons, ATP increased the NO production by the activation of P2X7 receptors [30]. In a formalin-induced model of orofacial pain, the P2 purinergic receptor antagonist pyridoxalphosphate-6-azophenyl-2′,4′-disulphonic acid (PPADS) decreased NOS expression in the trigeminal ganglia and Fos expression in the spinal trigeminal nucleus, which indicates a modulating effect of the purinergic signaling system on the nitroxidergic system [31]. ATP through activation of P2X receptors induced elevation of Ca^2+^ level which may increase NOS activity and subsequent NO release [32]. However, the interaction between NO and P2X receptors in the meningeal structures and trigeminal nerve system has not been described. 

In the present work, we (1) analyzed the effects of exogenous and endogenous NO on the activity of the peripheral afferents of the rat trigeminal nerve using isolated hemiskull preparation; (2) revealed the effects of the acute application and chronic administration of NO donors on the activity of the trigeminal nerve induced by TRPV1 and P2X receptor agonists; (3) studied the impact of TRPV1 and P2X receptors in the pro-nociceptive effects of NO; (4) elicited the mechanisms of NO effects on the activity of the trigeminal nerve induced by the activation of TRPV1; (5) assessed the degree of mast cell degranulation in the meninges of rats after the chronic administration of NG.

## 2. Results

### 2.1. Mechanical Sensitivity and Photophobia in Rats during Chronic Nitroglycerin (NG) Administration

**Figure 1:**
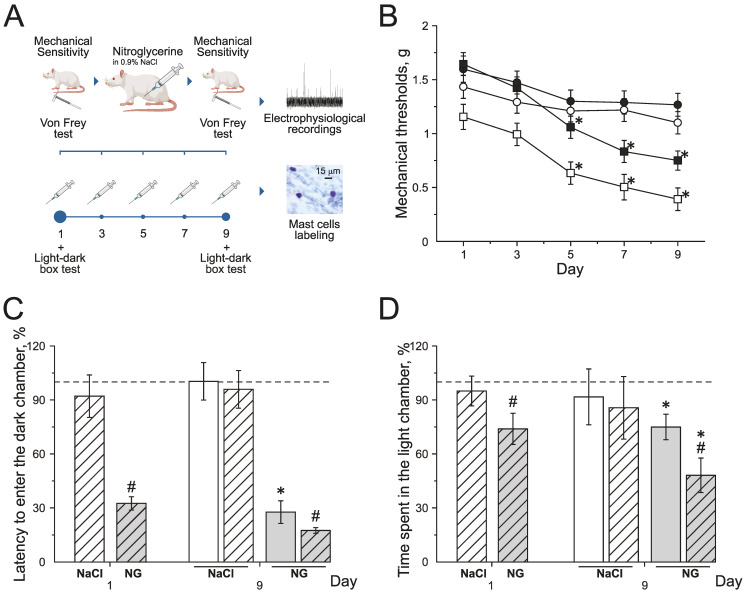
Effect of chronic nitroglycerin (NG) administration on mechanical sensitivity and photophobia. (**A**) Experimental scheme. (**B**) Mechanical withdrawal thresholds of rats before and two hours after the administration of NG (black and white squares) or vehicle (NaCl) (black and white circles). (**C**) Latency to enter the dark chamber and (**D**) time spent in the light chamber before (open columns) and two hours after (dashed columns) the administration of vehicle (white columns) or NG (grey columns) on the 1st and 9th day (in %) compared to the initial values taken as 100% and marked with a dotted line. * *p* < 0.05 compared to 100%; # *p* < 0.05 compared to the pre-injection level.

Chronic NG administration in rats induced the decrease of mechanical thresholds before and after injections. Mechanical thresholds were tested using von Frey filaments before (basal response) and 2 h after (post-injection response) NG administration every second other day for 9 days (Figure 1A,B). Basal mechanical sensitivity significantly increased by the 5th day of the experiment, and the mechanical threshold decreased to 1.3 ± 0.1 g (*n* = 16, *p* < 0.05) compared to the initial value—1.6 ± 0.1 g (Figure 1B; black squires). Post-injection responses significantly decreased after the 3rd injection of NG to 0.6 ± 0.1 g (*n* = 16, *p* < 0.05, Figure 1B; white squires) compared to the initial value—1.1 ± 0.1 g. NaCl injections in the control group did not change mechanical thresholds before or after injections during the experiment (Figure 1B; black and white circles).

Photophobia was assessed in the dark–light box using the total time spent in the light chamber and the time to the first entry into the dark chamber before and after the NG injection on the 1st and 9th days of the experiment (Figure 1C,D).

During the first day before the NG injection, the time to the first entry into the dark chamber was 39.1 ± 5.3 s (*n* = 16) and the total time spent in the light chamber—81.9 ± 6.4 s; these numbers were taken as 100% (*n* = 16, Figure 1C,D). The first injection of NG reduced the latency to enter the dark chamber to 32% (*p* < 0.05; Figure 1C grey dashed columns) and the time spent in light chamber decreased to 74% (*p* < 0.05, Figure 1D grey dashed columns).

On the 9th day of the experiment, before the NG injection, the time to the first entry into the dark chamber was 10.8 ± 3.6 s (27% of the initial value; Figure 1C grey columns) and the time spent in the light chamber was 62.3 ± 7.1 s (75 % of the initial values, *p* < 0.05; Figure 1D grey columns). Two hours after the NG injection, the latency to enter the dark chamber decreased to 17.5% (*p* < 0.05, Figure 1C grey dashed columns) and the time spent in the light box decreased to 48 % (*p* < 0.05, Figure 1D grey dashed columns).

NaCl injections did not change any parameters of behavior in the dark–light box test (Figure 1C,D white dashed columns). The obtained data demonstrated the development of allodynia and photophobia in rats with chronic NG administration. 

### 2.2. Effect of Endogenous and Exogenous Nitric Oxide on the Electrical Activity of the Trigeminal Nerve of Rats

**Figure 2:**
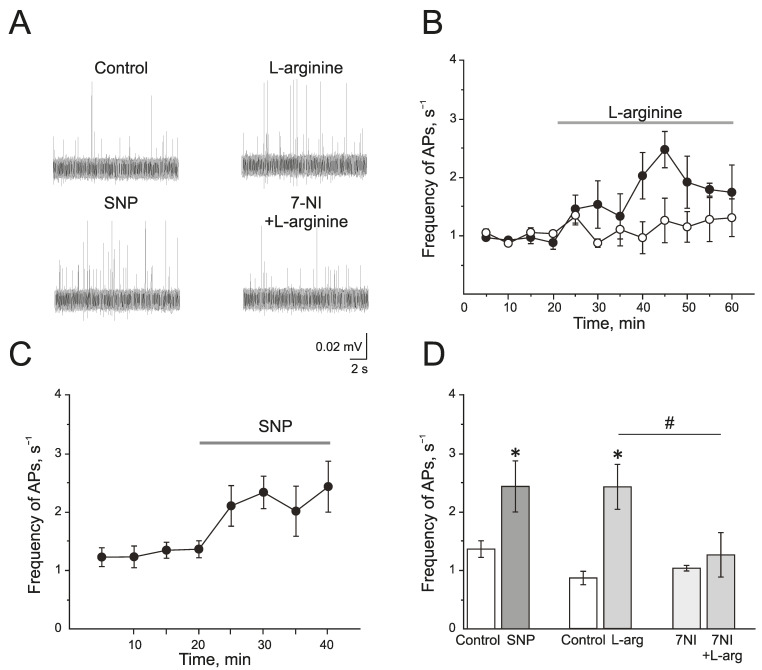
Endogenous and exogenous nitric oxide (NO) increases the electrical activity of rat trigeminal afferents. (**A**) Sample traces of AP in the trigeminal nerve of a rat in the control and after the application of L-arginine (300 μM), NO donor SNP (200 μM), and L-arginine in the presence of nNOS inhibitor, 7-nitroindazole (7NI, 1 mM); (**B**) The time-course of AP frequency after the application of L-arginine (black circles) and in the presence of 7NI (white circles); (**C**) The time-course of frequency of AP after the application of SNP; (**D**) Histogram showing the maximum frequency of AP per 5 min after the application of L-arginine, SNP, 7NI and 7NI + L-arginine. Mean ± SEM. * *p* < 0.05 compared to control values; # *p* < 0.05 compared to the effect of L-arginine as a control.

L-arginine (300 µM), a substrate of NOS, induced a significant increase in the action potential (AP) frequency from 0.77 ± 0.12 s^−1^ to 2.47 ± 0.31 s^−1^ by 25 min of administration (*n* = 4; *p* < 0.05; Figure 2A,B,D). Similar results but with faster development were obtained after the application of a NO donor—sodium nitroprusside (SNP, 200 µM). SNP increased the frequency of AP from 1.35 ± 0.14 s^−1^ to 2.32 ± 0.27 s^−1^ by 10 min and to 2.42 ± 0.43 s^−1^ by 20 min (*n* = 6; *p* < 0.05; Figure 2A,C,D).

A selective inhibitor of nNOS, 7-nitroindazole (7NI), at a concentration of 1 mM had no effects on the electrical activity of trigeminal nerve during 1 h of incubation and AP frequency was 1.03 ± 0.04 s^−1^ (*n* = 6). Subsequent application of L-arginine (300 µM) did not change the frequency of AP, which was 1.25 ± 0.37 s^−1^ in 25 min (*n* = 6; *p* > 0.05; Figure 2A,B,D). 

Therefore, the exogenous and endogenous NO synthesized by nNOS increases the electrical activity of the trigeminal nerve.

### 2.3. Interaction between NO and P2X3 Receptors in the Regulation of Electrical Activity of the Trigeminal Nerve of Rats

**Figure 3:**
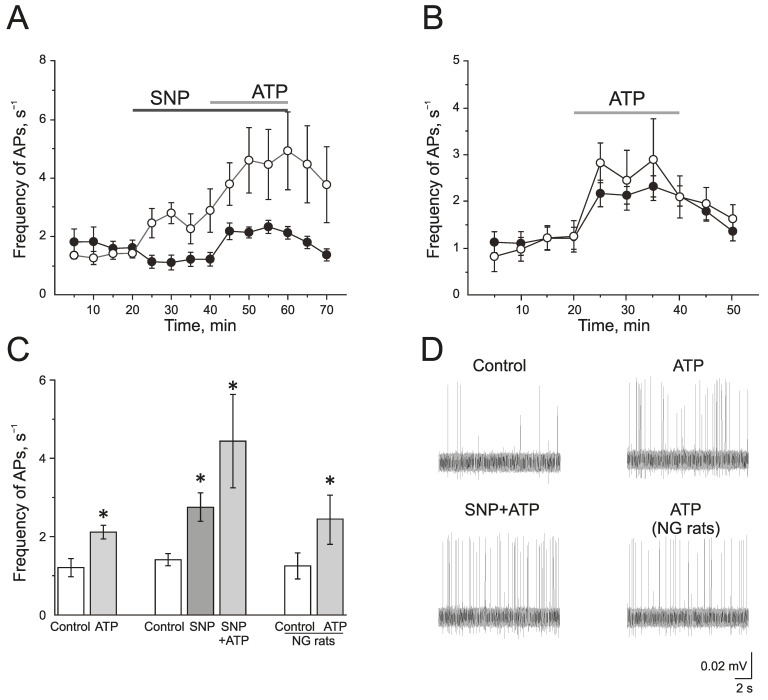
The role of NO in the pro-nociceptive action of ATP in the trigeminal nerve afferents. (**A**) The time-course of AP frequency during ATP (100 µM) application in the control group (black circles) and in the group after the application of the NO donor (SNP 200 µM, white circles); (**B**) The time-course of the AP frequency during the application of ATP in the control (black circles) and in the nitroglycerine (NG)-induced migraine model (white circles); (**C**) Histogram showing the maximum frequency of AP per 5 min in the control, after the application of ATP; SNP, ATP in the presence of SNP and the effect of ATP in the NG-induced migraine model group (NG rats); (**D**) Sample traces of AP in the trigeminal nerve in the control, after the application of ATP, application of ATP in the presence of SNP, and application of ATP in the NG-induced migraine model group (NG rats). Mean ± SEM. * *p* < 0.05.

To study the effect of the exogenous donor NO, SNP 200 μM, on the activity of P2X3 receptors, ATP was used at a concentration of 100 μM. In the control group, ATP (100 μM) significantly increased the frequency of AP from 1.20 ± 0.23 s^−1^ to 2.12 ± 0.18 s^−1^ by 10 min (*n* = 11; *p* < 0.05) and to 2.10 ± 0.21 s^−1^ by the 20 min application (*n* = 11; *p* < 0.05; Figure 3A,C,D).

The incubation of the hemiskull preparation in a solution containing SNP for 20 min increased the frequency of AP twofold from 1.41 ± 0.15 s^−1^ to 2.77 ± 0.35 s^−1^ by 10 min (*n* = 4; *p* < 0.05; Figure 3A,C,D). The subsequent application of ATP further elevated the frequency of AP up to 4.58 ± 1.11 s^−1^ by 10 min and to 4.90 ± 1.32 s^−1^ by 20 min (*p* < 0.05; Figure 3A,C). 

In the NG-induced migraine model, the frequency of AP in the trigeminal nerve afferents was 1.24 ± 0.33 s^−1^, which did not differ significantly from the control animal group. The application of ATP (100 µM) increased the frequency of AP similar to the control group (Figure 3B–D). The frequency of AP was elevated up to 2.44 ± 0.63 s^−1^ by 10 min and to 2.08 ± 0.44 s^−1^ by 20 min (*n* = 6; *p* < 0.05).

**Figure 4:**
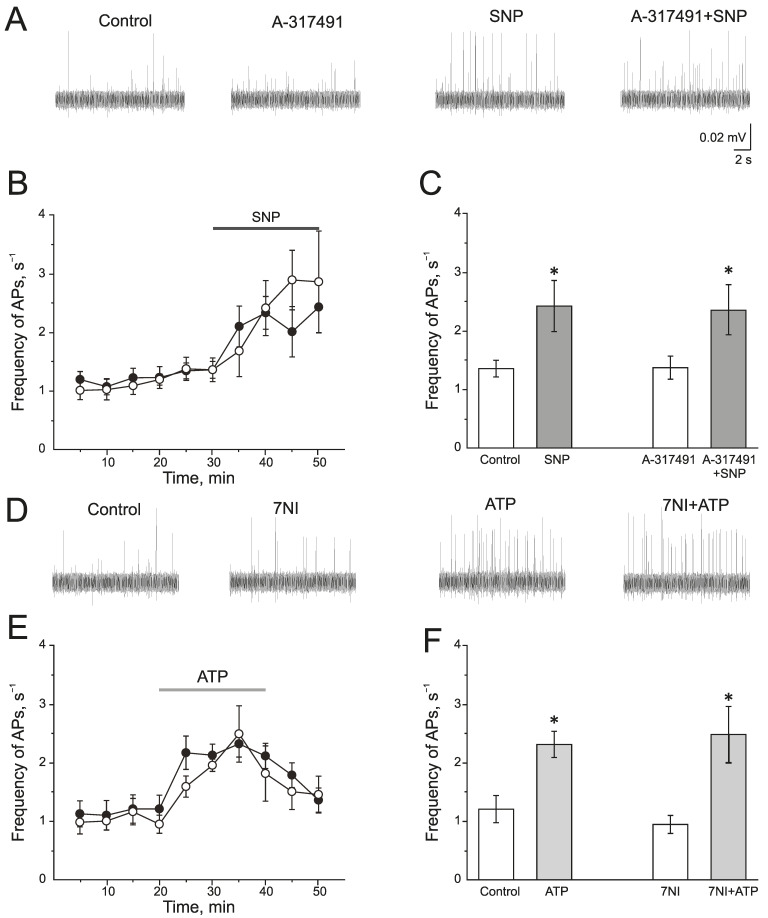
Interaction of NO and P2X3 receptors in rat trigeminal afferents (**A**) Sample traces of AP in the trigeminal nerve in the control or after the inhibition of P2X3 receptors by A-317491 (10 µM), after the application of the NO donor—SNP (200 µM) and A-317491 + SNP; (**B**) The time-course of AP frequency after the application of SNP in control (black circles) and after the inhibition of P2X3 receptors by A-317491 (white circles). (**C**) Histogram showing the maximum frequency of AP per 5 min in the trigeminal nerve after the application of SNP and A-31749 + SNP. (**D**) Sample traces of AP in the control, after the application of 7NI, ATP and 7NI + ATP; (**E**) The time-course of AP frequency after the application of ATP in the control (black circles) and in the presence of 7NI (white circles); (**F**) Histogram showing the maximum frequency of AP per 5 min after the application of ATP in the control and in the presence of 7NI. Mean ± SEM. * *p* < 0.05.

A selective P2X3 receptor antagonist A-317491 (10 µM) was used to identify the role of P2X3 receptors in the pro-nociceptive action of NO (Figure 4A–C). A-317491 did not change the frequency of AP, which was 1.19 ± 0.09 s^−1^ before and 1.37 ± 0.19 s^−1^ by 10 min of A-317491 application. Subsequent application of SNP increased the frequency of AP up to 2.40 ± 0.46 s^−1^ by 10 min (*n* = 4; *p* < 0.05) and to 2.85 ± 0.86 s^−1^ by 20 min (*n* = 4; Figure 4B,C). Therefore, the pro-nociceptive effect of SNP did not depend on the activation of P2X3 receptors.

There is evidence that the activation of P2X receptors increases NOS activity and the production of endogenous NO. To verify the impact of endogenously produced NO in the effects of ATP, nNOS was inhibited by 7NI (1 mM, 1 h). In this condition, ATP increases the frequency of AP to the same extent as in the control (Figure 4D–F). The initial frequency of AP was 0.94 ± 0.15 s^−1^ and ATP increased the frequency of AP up to 1.94 ± 0.11 s^−1^ (*n* = 4; *p* < 0.05) by 10 min, by 20 min—1.80 ± 0.47 s^−1^ (*n* = 4; Figure 4E).

Thus, the acute application of the exogenous NO donor, SNP, ex vivo as well as the chronic administration of NG in vivo does not reliably affect the intensity of pro-nociceptive action of ATP in the rat trigeminal nerve afferents.

### 2.4. Interaction between Nitric Oxide and TRPV1 in the Regulation of Action Potential Frequency in the Trigeminal Nerve of Rats

**Figure 5:**
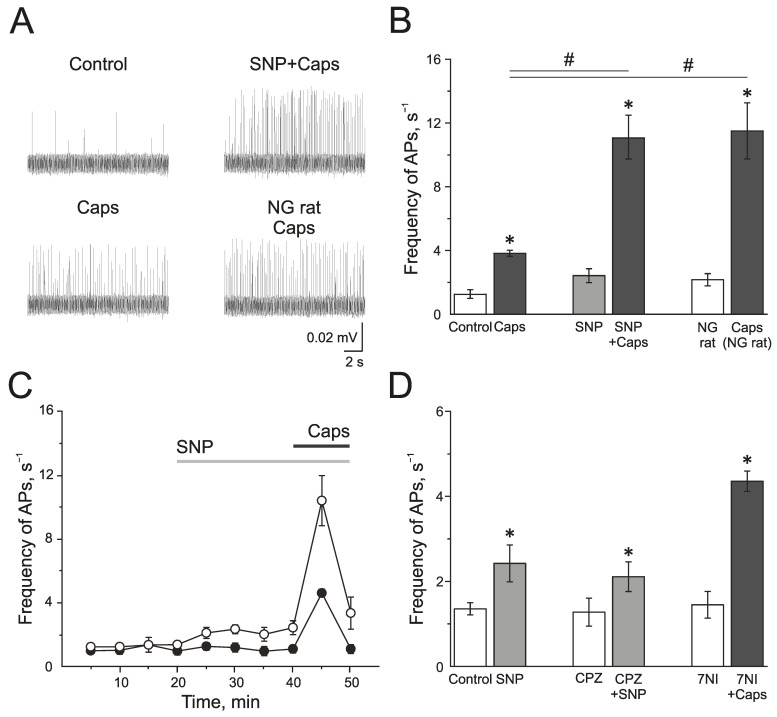
Effects of NO on activity of TRPV1 in the rat trigeminal afferents. (**A**) Sample traces of AP in the trigeminal nerve in controls, after the application of capsaicin (1 µM), capsaicin in the presence of NO donor, SNP (200 µM), and the application of capsaicin in NG-induced migraine model group. (**B**) Histogram showing the mean frequency of AP per 5 min in the trigeminal nerve after the application of capsaicin in the control, after application of the NO donor SNP and in the NG-induced migraine model group; (**C**) The time-course of frequency of AP after the application of capsaicin in the control (black circles) and after the application of SNP (white circles); (**D**) Histogram showing the mean frequency of AP per 5 min after the application of SNP in the control group, application of SNP after the inhibition of TRPV1 receptors by capsazepine (20 µM) and capsaicin after inhibition of nNOS by 7NI (1 mM). Mean ± SEM. * *p* < 0.05 (compared to the control); # *p* < 0.05 (compared to the effect of capsaicin in the control).

The TRPV1 receptor agonist capsaicin (1 µM) induced a strong and transient increase of electrical activity in the trigeminal nerve afferents in the control group from 1.28 ± 0.28 s^−1^ to 3.83 ± 0.17 s^−1^ by 5 min (*n* = 7; *p* < 0.05; Figure 5A,B). SNP increased AP frequency from 1.35 ± 0.14 s^−1^ up to 2.42 ± 0.43 s^−1^ by 20 min of incubation. Subsequent application of capsaicin (1 µM) significantly increased the electrical activity to 11.1 ± 1.36 s^−1^ (*n* = 6; *p* < 0.05; Figure 5A,B). The preparations from rats treated with NG capsaicin (1 µM) increased the frequency of AP from 2.16 ± 0.38 Hz to 11.50 ± 1.75 s^−1^ (*n* = 6; *p* < 0.05; Figure 5A,B). Therefore, NO promotes the activation of the trigeminal afferents by TRPV1 agonist capsaicin.

Next, we tested if TRPV1 activity impacted the pro-nociceptive effect of NO. Capsazepine did not affect the frequency of AP, and the further application of SNP increased spiking from 1.37 ± 0.33 s^−1^ to 2.11 ± 0.35 s^−1^ (*n* = 4; *p* < 0.05) by 10 min, and to 2.91 ± 0.61 s^−1^ by 20 min (*n* = 4; *p* < 0.05), which did not differ significantly from the effects of SNP in the control group (2.42 ± 0.43 s^−1^ by 20 min of incubation (*n* = 6; *p* < 0.05) (Figure 5D). Subsequent application of capsaicin after the administration of capsazepine and NO donor did not increase the frequency of AP, which confirms the inhibition of TRPV1 (frequency of AP was 2.50 ± 0.67 s^−1^).

Effects of capsaicin after the inhibition of nNOS by 7NI (1 mM) was the same as its effects in the control, and the frequency of AP increased from 1.45 ± 0.31 s^−1^ to 4.35 ± 0.24 s^−1^ (*n* = 4; *p* < 0.05; Figure 5D), which indicated that TRPV1 activation does not activate the endogenous NO production.

Thus, acute and chronic introduction of NO promotes the pro-nociceptive effects of the activation of TRPV1.

### 2.5. Mechanism of NO Action on TRPV1 in the Trigeminal Nerve Afferents 

**Figure 6:**
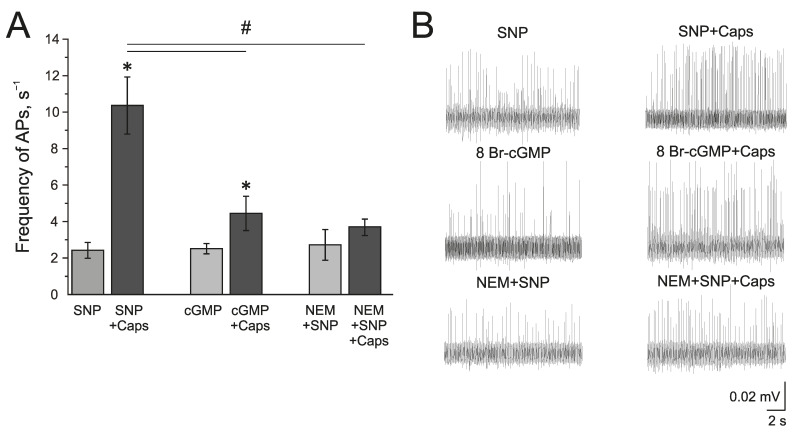
Mechanism of the modulating effect of NO on TRPV1 receptors. (**A**) Histogram showing the maximum frequency of AP per 5 min in the trigeminal nerve after the application of the NO donor SNP (200 μM) and capsaicin (1 μM) in the presence of SNP; cGMP analogue—8-Br-cGMP (500 μM) and capsaicin in the presence of 8-Br-cGMP; N-Ethylmaleimide, (NEM, 25 μM) together with SNP and capsaicin in the presence of NEM + SNP. (**B**) Sample traces of AP in the trigeminal nerve after the application of SNP and SNP + Caps; 8-Br-cGMP and 8-Br-cGMP + Caps; NEM and NEM + SNP + Caps. Mean ± SEM. * *p* < 0.05 (compared to SNP or cGMP respectively); # *p* < 0.05 (compared to the effect of capsaicin + SNP).

The known mechanisms of the NO action on TRPV1 can be related to the sGC/cGMP/PKG-dependent pathway or direct S-nitrosylation of the TRPV1 protein. The membrane-penetrating cGMP analog 8Br-cGMP (500 µM), similar to SNP, increased the frequency of AP twofold from 1.09 ± 0.05 s^−1^ to 1.76 ± 0.33 s^−1^ by 10 min and to 2.51 ± 0.27 s^−1^ by 20 min (*n* = 4, *p* < 0.05) (Figure 6A,B). Further capsaicin (1 µM) application increased the frequency of AP up to 4.44 ± 0.93 s^−1^, which is significantly lower than the effects of capsaicin after incubation in SNP (Figure 6A).

The inhibitor of S-nitrosylation—N-Ethylmaleimide, NEM (25 µM), prevented the NO-induced increase of the capsaicin effect in the rat trigeminal nerve afferents (Figure 6A). After the incubation of the hemiskull preparation in a solution containing NEM + SNP, the application of capsaicin (1 µM) did not induce the elevation of AP frequency (2.72 ± 0.84 s^−1^ to 3.67 ± 0.43 s^−1^ (*n* = 4; Figure 6A). Therefore, we can propose that the positive effects of NO on TRPV1 are mediated by S-nitrosylation.

### 2.6. Effect of Chronic Administration of NG on Mast Cells Degranulation in Rat Meninges 

**Figure 7:**
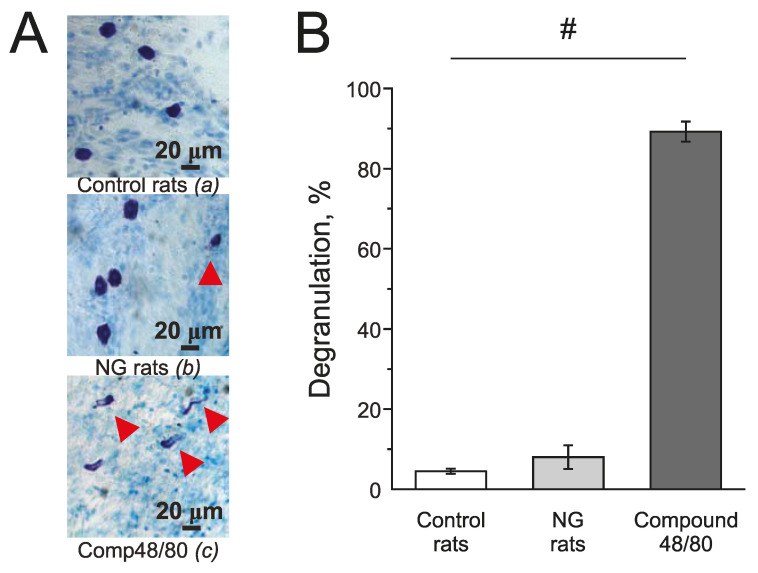
Effect of chronic nitroglycerine administration on mast cell degranulation in rat meninges. (**A**) Toluidine Blue staining of the meninges in the control (**a**) (*n* = 5); in the NG-induced migraine model group (NG rats; *n* = 4); (**b**); after exposure to Compound 48/80 in the control (10 mg/mL; 30 min; *n* = 4) (**c**). Notice red arrows indicating degranulated mast cells. (**B**) Histograms showing the percent of degranulated mast cells in the control group, model group, and after Compound 48/80. Mean ± SEM. # *p* < 0.05.

Mast cells in the meninges were stained with Toluidine blue. Compound 48/80 (10 mg/mL) induced massive degranulation of mast cells up to 89.21 ± 2.53% (*n* = 4; Figure 7(Ac),B) and was used as a positive control. In the control group, the number of degranulated mast cells was 4.5 ± 0.64 % (*n* = 5; Figure 7(Aa),B) and in the NG-induced migraine model group—7.02 ± 2.92 % (*n* = 4; Figure 7(Ab),B).

## 3. Discussion

NO is a gasotransmitter involved in the pathogenesis of migraine; it can be produced in the meninges by eNOS, expressed in the endothelium of cerebral vessels (including the MMA), and by nNOS, localized in the trigeminal afferents [13,14,15,33]. To analyze the effects of NO on the activity of the trigeminal nerve afferents, we used a rat hemiskull preparation that allows us to record the nociceptive activity directly at the site of its occurrence [34]. An exogenous NO donor, SNP, caused an increase in the electrical activity of the trigeminal nerve already by 10 min, which indicates its pro-nociceptive effect, confirming previous data [14,35]. Additionally, the substrate of NO synthesis, L-arginine, similarly increased the frequency of AP but more slowly—by 25 min application, and its effect depended on NOS activity, since a nNOS inhibitor prevented the effects of L-arginine.

Our findings are consistent with those of other studies that demonstrated the main role of nNOS in central and peripheral nociception; however, there were no data proving the direct effects of NO and L-arginine on the meningeal afferents. Previous data showed the involvement of NO in the formalin-induced orofacial pain model where nNOS expression in the trigeminal ganglion neurons and in the trigeminal nucleus caudalis was increased [36]. The endogenous stimuli of NOS include proinflammatory cytokines, CGRP, and an increase in intracellular calcium concentration due to the activation of NMDA receptors [13], whose functional role was shown in the neurons of the trigeminal ganglion both in the soma and in the endings of the trigeminal nerve-innervated meninges [37]. In addition, cortical spreading depression (CSD), which is considered the electrophysiological correlate of migraine with aura, may contribute to a NO release that causes not only vascular but also neuronal effects [13,38,39].

Nitroglycerin (NG) as a NO donor is widely used both to model an episodic migraine with a single injection and chronic migraine with multiple injections [40,41,42,43,44]. Chronic administration of NG in our study caused the development of allodynia and photophobia, which confirms the development of symptoms of migraine in rats. A single injection of NG to rodents (10 mg/kg) causes the sensitization of both primary afferent (trigeminal ganglion) and central trigeminocervical neurons [43,45,46]. Such types of sensitization may be caused by the release of pro-inflammatory agents participated in the pathogenesis of migraine such as PACAP, BDNF [47], and CGRP [48,49,50,51,52]. In addition, NG caused an increase in the nNOS expression in the trigeminal nucleus caudalis (TNC) [53], in the dura and trigeminal ganglia [54,55,56], whereas the nNOS inhibitor 7NI prevented the NG-induced activation of structures involved in trigeminal nociception [44,54,57]. No changes in the thresholds and parameters of CSD in the rats’ somatosensory cortex were observed after the administration of NG that confirms the predominantly peripheral and brain stem mechanisms of its action [38]. Despite a significant amount of data related to the mechanisms of NG-induced sensitization of nociceptive pathways, the role of P2X3 and TRPV1 receptors—the key pain transducers expressed in the meningeal nociceptors—in the effects of NO has not been studied.

According to the purinergic theory of migraine, ATP, on the one side, mediates the vascular changes during a migraine attack [58]. On the other side, ATP stimulates directly the primary afferents located along the cerebral vessels through P2X3 receptors, which are expressed both in the afferent endings and in the trigeminal ganglion neurons [17,25,27,59,60,61,62]. In addition, the pro-nociceptive effect of ATP is aggravated by the degranulation of meningeal mast cells followed by the release of serotonin, which has its own neuronal effects through 5-HT3 receptors [25,61,63,64]. The interaction among P2X3 receptors, CGRP, and NO was proposed in migraine-associated signaling cascades [15]. There is an evidence of the co-localization of nNOS with P2X5 and P2X2 receptors in different parts of the brain and that activation of ATP receptors can induce NO production and enhance nNOS expression [31,65].

In our study, the inhibition of P2X3 receptors did not change the pro-nociceptive effects of the SNP in the rat meninges. In addition, the increase of trigeminal nerve activity due to the application of ATP was not dependent on the endogenous synthesis or on the exogenous administration of NO. Moreover, in the model of chronic migraine in rats, the rate of mast cell degranulation did not change compared to the control. Thus, in our experiment’s conditions, both short-term incubation in the SNP or the chronic administration of the NG did not affect the pro-nociceptive action of ATP in the meningeal afferents.

TRPV1 receptors are considered to be one of the most important components in the pathogenesis of migraine [26,66,67,68]. TRPV1 can be activated by various stimuli, including temperature (~42 °C), pH, and a wide range of both endogenous and exogenous compounds including capsaicin [67]. TRPV1 channels are actively expressed in the structures involved in the nociceptive process, such as DRG neurons, the brainstem, peptidergic and non-peptidergic C-fibers and some Aδ-fibers [67]. TRPV1 expression was shown in the meninges and sensory afferents using immunohistochemical methods [69,70].

The activation of TRPV1 is able to activate the meningeal nociceptors directly and promote CGRP release from sensory nerve endings [66]. TRPV1 can be activated in the meninges during migraine attacks, and the expression of TRPV1 changes during CSD [11]. TRPV1 are extremely sensitive to various chemical modifications. Thus, oxidizing agents are able to positively modulate (sensitize) the TRPV1 channel [71] through the covalent modification of cysteines [72]. Sulfhydryl agents such as NaHS [73] or DTT directly modulate the channel activity at sites located both extracellularly and within cytoplasmic domains [74]. Previously, it was shown that exogenous or endogenous NO can both positively and negatively modulate TRPV1 activity [18,19,20,55,75]. However, the interaction of NO and TRPV1 in the meningeal afferents has not been studied. In our experiments, the TRPV1 agonist capsaicin (1 μM) caused a significant and short-term increase in the spike frequency, consistent with previous published data [68,70,73], and this effect did not change after the inhibition of nNOS. However, acute or chronic elevation of NO significantly augmented the pro-nociceptive effect of capsaicin.

sGC is one of the main targets of NO [76], and in the trigeminal nerve afferents, the pro-nociceptive effect of NO was prevented by the inhibition of sGC [14]. Therefore, we studied the effect of the membrane-penetrating analogue of cGMP on TRPV1 activation. 8-BrcGMP caused an increase in the frequency of spikes similar to the NO donor SNP. The subsequent application of capsaicin caused an increase in the frequency of APs, similar to its effect in the control. Apparently, the increase of the TRPV1 response in the presence of NO is not associated with the sGC/cGMP/PKG signaling pathway.

Another common mechanism of NO action is direct chemical modification of proteins—S-nitrosylation, which was shown for a number of proteins, including NMDA receptors, Na^+^, K^+^ and Ca^2+^ channels, mitochondrial enzymes, and transcriptional factors [77]. In particular, NO can modulate the activity of TRPV1 by S-nitrosylation of extracellular cysteines Cys616 and Cys621 located in the pore loop of TRPV1 channels [19], which was suggested as a mechanism for nociceptive NO effects in DRG cells [20]. In the current study, we used an inhibitor of S-nitrosylation, NEM, which is a specific alkylating agent of cysteine sulfhydryls; it covalently modifies the sulfhydryl groups of proteins, thereby preventing subsequent S-nitrosylation [78]. It was shown that NEM completely prevented the increase in trigeminal nerve activity by capsaicin applied after the preincubation in SNP, indicating the role of S-nitrosylation in the effects of NO on TRPV1.

## 4. Materials and Methods

The experiments were performed on male Wistar rats of age P40–45 (P—day of birth). All experimental protocols were performed in accordance with the European Community Council Directive of 22 September 2010 (2010/63/EEC) and approved by the Local Ethical Committee of Kazan Federal University (protocol 33 dated 25 November 2021). All efforts were made to minimize the number of animals used in the experiments.

### 4.1. Behavioral Analysis in the Rat Migraine Model NG

Nitroglycerin (NG, Ozon Pharmaceuticals, Zhigulevsk, Russia) was used to create a model of chronic migraine in rats with testing the mechanical sensitivity and photophobia [38,79]. NG (10 mg/kg in 0.9% NaCl solution) was repeatedly administered intraperitoneally (IP) [38,79], five times every second day for nine days (days 1, 3, 5, 7, and 9). Mechanical hypersensitivity was measured 2 h before (pre-injection/basal response) and 2 hours after (post-injection response) NG injections [80]. The development of photophobia was assessed using the dark–light chamber test before and after the first and last injections of NG. In the control group, NaCl was introduced as a vehicle. All behavioral experiments were conducted at the same time and started at approximately 10 a.m.

#### 4.1.1. Von–Frey Test

Mechanical sensitivity was assessed with a series of calibrated Von–Frey filaments (Ugo Basile, Gemonio, Italy) with a range of 0.008 to 8 g of the target force, which corresponds to 2.53–61.7 g/mm^2^ pressure. At 30 min before the test, the rat was placed in an individual transparent box with a mesh floor. The mechanical withdrawal thresholds were determined according to the up-and-down method [38,40,81]. Von–Frey filaments were applied by approaching the plantar surface of the paw from the underside of the mesh stand. We always started by testing with a 0.4 g (3.61 g/mm^2^) filament. In all cases, the tip of the filament was pressed against the plantar surface of one hind paw maintained for 1–3 s. A response is defined as withdrawal, shaking, or licking of the paw. In the absence of a response, a heavier filament (up) was tried after 10 s, and in the presence of a response, a lighter filament (down) was tested. This pattern was followed for a maximum of four filaments following the first response.

#### 4.1.2. Light–Dark Transition Test

The Light–Dark Transition test was used to measure photophobia [82]. The light–dark box consists of two equally sized chambers: one illuminated (150 lux) and one darkened (1–2 lux) with a size of 40 × 20 × 40 cm/each connected with a passageway 7 × 7 cm (OpenScience, Moscow, Russia) equipped with a video system Sony SSC-G118 (Tokyo, Japan). The rats were placed in the light compartment and allowed to explore the apparatus for 3 min. We measured the latency to enter the dark chamber and the time spent in the light chamber.

### 4.2. Electrophysiology

To study the electrical signals arising in the peripheral processes of the trigeminal nerve, we used a preparation of an isolated rat skull. The skull was cleaned from the surrounding tissues, divided into two halves. The brain was then carefully removed from half of the skull, leaving the dura mater intact [68,83]. The processes of the trigeminal nerve (nervus spinosus), innervating the middle meningeal artery, were preserved in the dura mater of the brain.

Rat hemiskull preparation was constantly perfused with Krebs solution of the following composition (in mM): NaCl 120, KCl 2.5, CaCl_2_ 2, MgCl_2_ 1, glucose 11, Na_2_HPO_4_ 1, NaHCO_3_ 24, pH = 7.2–7.4 with constant aeration with carbogen (O_2_ 95%/CO_2_ 5%). The recording electrodes were made from borosilicate glass; the diameter of the electrode tip was around 150 µm. Under visual control, the peripheral process of the trigeminal nerve, nervus spinosus (V1 branch of the trigeminal nerve) was isolated from the dura mater and placed in a glass-recording electrode. The spontaneous or evoked by agonists action potentials (AP) in the trigeminal nerve were recorded using a DAM 80 amplifier (World Precision Instruments, Sarasota, FL, USA).

The following drugs were used during the study: NOS substrate, L-arginine; donor NO, Sodium nitroprusside (SNP), ATP (all drugs from Sigma-Aldrich, St. Louis, MO, USA) were prepared immediately before usage and dissolved in water. Inhibitor of neuronal NOS—7-nitroindozole, 7NI, agonist and antagonist of TRPV1-receptor—Capsaicin and Capsazepine were dissolved in DMSO (all drugs from Sigma-Aldrich). Antagonist of P2X3 receptor A-317491 sodium salt hydrate (Sigma-Aldrich), Inhibitor of S-nitrolyzation—N-Ethylmaleimide (NEM, Serva, Heidelberg, Germany), cGMP analog—8-Bromoguanosine 3′,5′-cyclic monophosphate sodium salt (8-Br-cGMP, Sigma-Aldrich) were dissolved in water.

The signals were digitized on a PC using an NI PCI6221 board (National Instruments, USA). The signals were visualized and analyzed using WinEDR v.3.2.7 software (University of Strathclyde, UK). The control for comparison in each experiment served as a five-minute segment of the recording before the filing of the test substance.

### 4.3. Toluidine Blue Staining of Meningeal Mast Cells

Mast cell degranulation was assessed using the histological method of staining for the hemiskull with toluidine blue [84,85]. Intact skulls of control and migraine model rats were placed in paraformaldehyde (4% solution) for 12 h. Before isolating the meninges, the skulls were washed in a phosphate-buffered saline solution of the following composition (mM): 137 NaCl, 2.7 KCl, 10 Na_2_HPO_4_, 1.8 K_2_HPO_4_. The isolated meninges were fixed on a glass slide. Staining with toluidine blue lasted 10 min, and then the fixed preparations were washed with distilled water and dehydrated with ethyl alcohol (95–99%). Images were acquired with 20× magnification. The degree of degranulation was assessed visually; the calculation was carried out in the percentage of the total number of cells (at least 100).

### 4.4. Data Analysis

Statistical analysis was performed using Origin Pro 2018 (OriginLab Corporation, Northampton, MA, USA), Matlab v. 8.2 (The MathWorks, Natick, MA, USA) software. AP frequency was calculated as the number of APs per 300 s (s^−1^). The normality of the sample distribution was determined using the Kolmogorov–Smirnov test and the Shapiro–Wilk test. For data with a normal distribution, the significance of differences was assessed using a paired *t*-test for related samples and two sample *t*-test for independent samples. For data with a non-normal distribution, the Mann–Whitney test was used. Differences were considered statistically significant at *p* < 0.05; n indicates the number of animals. In the text, data are presented as mean and SE, where SE is the standard error.

## 5. Conclusions

In conclusion, our study demonstrated that exogenously and endogenously produced NO exhibits pro-nociceptive firing in the rat meningeal afferents of the trigeminal nerve and these effects were not dependent on the activation of P2X3 or TRPV1 receptors. At the same time, acute or chronic (nitroglycerine-induced migraine rat model) NO administration increased the pro-nociceptive effects of capsaicin, which may be associated with S-nitrosylation of TRPV1 or other targets impacting capsaicin-evoked firing. This mechanism can underlie the sensitization of the trigeminal afferents in a model of migraine induced by the chronic administration of NG. In this regard, TRPV1 receptors can be considered as an attractive target for drug intervention in chronic migraine.

## Data Availability

The data used to support the findings of this study are available from the corresponding author upon request.
